# *BMC Biotechnology* reviewer acknowledgement 2015

**DOI:** 10.1186/s12896-016-0245-1

**Published:** 2016-02-12

**Authors:** Dafne Solera

**Affiliations:** BioMed Central, Floor 6, 236 Gray’s Inn Road, London, WC1X 8HB UK

## Abstract

The editors of BMC Biotechnology would like to thank all our reviewers who have contributed to the journal in Volume 15 (2015).

**Sirajunnisa Abdul Razack**

India

**Sabu Abdulhameed**

India

**Robert Abramovitch**

USA

**Warish Ahmed**

Australia

**Christian Ahrens**

Switzerland

**Hal Alper**

USA

**T. R. Anish**

USA

**Wataru Aoi**

Japan

**Klaus-J. Appenroth**

Germany

**Ana C. M. Arisi**

Brazil

**Ranjana Arya**

India

**Stéphane Aymerich**

France

**Jongyoun Baik**

USA

**Pedro Baptista**

Spain

**Neil Barclay**

UK

**Karl Bayer**

Austria

**Frank Bedon**

Australia

**Evguenia Bekman**

Portugal

**Martina Bellasio**

Austria

**Harry Beller**

USA

**Isabelle Benoit**

Netherland

**Aleš Berlec**

Slovenia

**Maurizio Bettiga**

Sweden

**Silvia Bidarra**

Portugal

**Robert Bischof**

Austria

**Pernilla Bjerling**

Sweden

**John Blackwood**

UK

**Lars Mathias Blank**

Germany

**Andreas Bommarius**

USA

**Joseph Boothe**

Canada

**Massimiliano Borgogna**

Italy

**Nicole Borth**

Austria

**José Bragança**

Portugal

**David Brindley**

Singapore

**Mike Brisco**

Australia

**Jochen Büchs**

Germany

**Andreas Buehlmann**

Switzerland

**Matthias Bureik**

China

**Thomas Burmeister**

Germany

**Ornella Calderini**

Italy

**Pinar Calik**

Turkey

**Andrea Camattari**

Singapore

**Jijuan Cao**

China

**Hyung Joon Cha**

South Korea

**Mao Xiang Chen**

UK

**Rumei Chen**

China

**Tomohiro Chiba**

Japan

**Bijan Choudhury**

India

**Se Chul Chun**

South Korea

**Donatella Cimini**

Italy

**Dennis Claessen**

Netherland

**Martin Clynes**

Ireland

**Uli Commandeur**

Germany

**Tony Conner**

New Zealand

**Olga Correa**

Argentina

**Casey Crooks**

USA

**Jian Dong Cui**

China

**David Culley**

USA

**Tamás Dalmay**

UK

**Paul Daly**

UK

**Pascale Daran-Lapujade**

Netherland

**Mark Davis**

USA

**Noushin Davoudi**

Iran

**Liziane Maria De Lima**

Brazil

**Donatella De Pascale**

Italy

**Sandra De Weert**

Netherland

**Tigst Demeke**

Canada

**Vipin Deo**

Japan

**Leo Despaux**

USA

**Luigi Di Costanzo**

USA

**Isabel Dias**

Portugal

**Adiphol Dilokpimol**

Netherland

**Dino Dissel**

Netherland

**Jinhua Dong**

Japan

**Giovanna Donnarumma**

Italy

**Sarah E. F. D’Orazio**

USA

**Martin Dragosits**

Austria

**Dion Du Plessis**

South Africa

**Nishant Dwivedi**

USA

**Colin Eady**

New Zealand

**Brigitta Ebert**

Germany

**Lothar Eggeling**

Germany

**Abdellatif El M’Selmi**

France

**Dominic Esposito**

USA

**María Julia Estrella**

Argentina

**Thaddeus Ezeji**

USA

**Fan Fan**

USA

**Zhiliang Fan**

USA

**Phillip Farabaugh**

USA

**Jeanni Fehrsen**

South Africa

**Hugo Fernandes**

Portugal

**Tiago Fernandes**

Portugal

**John J. Finer**

USA

**Ayelet Fishman**

Israel

**Hans-Lothar Fuchsbauer**

Germany

**Manuel Fuentes**

Spain

**Alexander Fulton**

Germany

**Brigitte Gasser**

Austria

**Elena Geiser**

Germany

**Yoram Gerchman**

Israel

**Aron Geurts**

USA

**Nasser Ghaemi**

Iran

**M. Ghosh**

India

**Paula Giardina**

Italy

**Rajanish Giri**

India

**Graciela Glikmann**

Argentina

**Ee-Been Goh**

USA

**Billie Gould**

Canada

**Catherine Grgicak**

USA

**Ewa Grzebelus**

Poland

**Rani Gupta**

India

**Taekwang Ha**

Denmark

**Henning Hansen**

Denmark

**Kim Hebelstrup**

Denmark

**Êlke Heine-Dobbernack**

Germany

**Olivier Henry**

Canada

**Pablo Hernández**

Spain

**Hirofumi Hirai**

Japan

**Caroline Hoemann**

Canada

**Raimund Hoffrogge**

Germany

**Barbara Hohn**

Switzerland

**Hiroyuki Honda**

Japan

**Thomas Horbett**

USA

**David P. Horvath**

USA

**Huang Huang**

USA

**Michael Hust**

Germany

**Taisen Iguchi**

Japan

**Masahito Ikawa**

Japan

**Masashi Iwanaga**

Japan

**Grzegorz Janusz**

Poland

**Clark Jeffries**

USA

**Jingfang Ju**

USA

**Yi-Hsu Ju**

Taiwan

**Vipin Kalia**

India

**Héla Kallel**

Tunisia

**Masamichi Kamihira**

Japan

**Angelos K. Kanellis**

Greece

**Han Chang Kang**

USA

**Sang-Moo Kang**

USA

**Ajit Kanwal**

India

**Levente Karaffa**

Hungary

**Mythreye Karthikeyan**

USA

**Tatsuya Kato**

Japan

**Arvind Kayastha**

India

**Todd Kelley**

USA

**Nawazi Khan**

USA

**Chang Sup Kim**

South Korea

**Haeng-Hoon Kim**

South Korea

**Ho Min Kim**

South Korea

**Hyun Woo Kim**

South Korea

**Jong-Won Kim**

South Korea

**Sang Jick Kim**

South Korea

**Yeon-Gu Kim**

South Korea

**Stephan Kirchmaier**

Germany

**Heath Klock**

USA

**Tie Koide**

Brazil

**Jens Kossmann**

South Africa

**Mauri Kostiainen**

Finland

**Harald Kranz**

Germany

**László Kredics**

Hungary

**Jonas Kügler**

Germany

**Abhishek Kumar**

Germany

**Guruprasad Kuntamallappanavar**

USA

**Sang-Soo Kwak**

South Korea

**Kyu Kwon**

South Korea

**Soonjo Kwon**

South Korea

**Pavel Kyslík**

Czech Republic

**Rup Lal**

India

**Marina Laura**

Italy

**Denis Leclerc**

Canada

**Ruifang Li**

China

**Jiazhang Lian**

USA

**Shuli Liang**

China

**Junita Liebenberg**

South Africa

**Melissa Lin**

Singapore

**Ruijie Liu**

China

**Xiaolin Liu**

China

**Yajun Liu**

China

**Zewen Liu**

China

**Zoachang Liu**

China

**Verena Lohr**

Germany

**Liam Longo**

Israel

**Catia Giovanna Lopresto**

Italy

**Marina Lotti**

Italy

**Xiaochu Lou**

USA

**Arianna Lovato**

Italy

**Hua Lu**

USA

**Xiaofeng Lu**

USA

**Roland Ludwig**

Austria

**Taina Lundell**

Finland

**Hong Luo**

USA

**Yinggang Luo**

China

**Jill Madine**

UK

**François Madore**

Canada

**Ivan G. Maia**

Brazil

**Giuseppe Manco**

Italy

**Giuseppe Mandolino**

Italy

**Maheswaran Mani**

India

**Ben Mans**

South Africa

**Francois Maree**

South Africa

**Helena Maresová**

Czech Republic

**Celestina Mariani**

Netherland

**Nelson Marmiroli**

Italy

**Enzo Martegani**

Italy

**Dirk E. Martens**

Netherland

**Ludmila Martinkova**

Czech Republic

**Albino Martins**

Portugal

**Autar Mattoo**

USA

**Steve Maxwell**

USA

**Mark Mccann**

New Zealand

**Praveen Mehta**

India

**Wenzhao Meng**

USA

**Severine Menoret**

France

**Joachim Messing**

USA

**Yungen Miao**

China

**Michael Miller**

USA

**Yoshiko Minami**

Japan

**Nuno Mira**

Portugal

**Bruno Miroux**

France

**Narayan Chandra Mishra**

India

**Saroj Mishra**

India

**Gisele Monteiro**

Brazil

**Emilio Montesinos**

Spain

**Kuntal Mukerjee**

USA

**Ulrike G. Munderloh**

USA

**Jolanda Munster**

UK

**Sean Vincent Murphy**

USA

**Nafiseh Nafissi**

Canada

**Hiroyuki Nakai**

USA

**Hiroshi Nakayama**

Japan

**Kee Woei Ng**

Singapore

**Jean Marc Nicaud**

France

**Ken-Ichi Nishijima**

Japan

**Yuval Nov**

Israel

**Cory Nykiforuk**

Canada

**Kevin O’Connor**

Ireland

**Rosa Marıa Oliart-Ros**

Mexico

**Chalermporn Ongvarrasopone**

Slovenia

**Gary R. Ostroff**

USA

**Seung Pil Pack**

South Korea

**Hongbo Pang**

USA

**Nádia Parachin**

Brazil

**Sheldon J. Park**

USA

**Sungjo Park**

USA

**Ermenegilda Parrilli**

Italy

**Siavash Partow**

Canada

**Ashok Patel**

India

**Basant Patel**

India

**Leslie Pedrioli**

UK

**Matthias Peipp**

Germany

**Yingjie Peng**

USA

**James Petrie**

Australia

**Mauro Petrillo**

Italy

**Jitka Petrlova**

Sweden

**Rogério Pezato**

Brazil

**Steven Poelzing**

USA

**Matt Posewitz**

USA

**James Preston**

USA

**Russ Price**

USA

**Roland Prielhofer**

Austria

**Holger Puchta**

Germany

**Qiudeng Que**

USA

**Marko Radic**

USA

**Zeljko Radulovic**

USA

**Qasim Rafiq**

UK

**Arun Rajkumar**

Denmark

**Bruce Ramsay**

Canada

**Gurinderjit Randhawa**

India

**Nicolas Rasche**

Germany

**Norman A. Ratcliffe**

UK

**Girish Ratnaparkhi**

India

**Ana Filipa Rodrigues**

Portugal

**Carlos Rodrigues**

Portugal

**Susana Rodriguez-Couto**

Spain

**Gertrudis Rojas**

Cuba

**Antonio Roldao**

Portugal

**Carlos Romero**

USA

**Nancy Roosens**

Belgium

**Stefan Rose-John**

Germany

**Daniele Rosellini**

Italy

**Akihide Ryo**

Japan

**Xavier Saelens**

Belgium

**Bhaskar Saha**

India

**Masakiyo Sakaguchi**

Japan

**Christiane Salgado**

Portugal

**Ana Marisa Salgueiro**

Portugal

**Maurilio Sampaolesi**

Belgium

**Sébastien Sart**

Belgium

**Chiara Schiraldi**

Italy

**Lutz Schmitt**

Germany

**Bernhard Seiboth**

Austria

**Seung-Oh Seo**

USA

**Davide Seruggia**

Spain

**Luisa Seuanes Serafim**

Portugal

**Frederick Sheedy**

Ireland

**Hamid Shegarfi**

Noeway

**Xiaolin Shen**

China

**Zhicheng Shen**

China

**Abhay Shendye**

USA

**Oded Shoseyov**

Israel

**Irina Simões**

UK

**Raushan Singh**

USA

**Yogendra Singh**

India

**Gordon Smyth**

Australia

**Jonathan Snow**

USA

**Ola Söderberg**

Sweden

**Ulrich Soltmann**

Germany

**Oliver Spadiut**

Austria

**Alagarsamy Srinivasan**

USA

**Arun Srivastava**

USA

**Sanjeeva Srivastava**

India

**Igor Stagljar**

Canada

**Joseph Stains**

USA

**Piotr Stefanowicz**

Poland

**Matthias Steiger**

Austria

**Antonietta Stellavato**

Italy

**Liudmila Stepanova**

Russian Federation

**C. Neal Stewart**

USA

**Francesca Storici**

USA

**Gerald Striedner**

Austria

**Piruthivi Sukumar**

India

**Liangliang Sun**

USA

**Kamilla Swiech**

Brazil

**György Szittya**

Hungary

**Rouzbeh Taghizadeh**

USA

**Tatsuya Takemoto**

Japan

**Ralf Takors**

Germany

**Irene Tan**

Malaysia

**Ryouichi Tanaka**

Japan

**Yong Tang**

Canada

**Sheng-Ce Tao**

China

**Yosuke Tashiro**

Japan

**Christof Taxis**

Germany

**Alexander Ter Beek**

Netherland

**Roger Thilmony**

USA

**Xu Tian**

USA

**Viktorija Tomic**

Slovenia

**Belinda Townsend**

UK

**Yoshiaki Tsuji**

USA

**Aysel Ugur**

Turkey

**Jeroen P. Van Dijk**

Netherland

**Mirinda Van Kleef**

South Africa

**Petrus Van Zyl**

South Africa

**Anikó Várnai**

Norway

**Ajay Vashisht**

USA

**Vinod Vathipadiekal**

USA

**Phanindra Velisetty**

USA

**Hans Visser**

Netherland

**Bernd Voedisch**

Germany

**Jianhua Wang**

China

**Yimeng Wang**

USA

**Lawrence Wangh**

USA

**Hajime Watanabe**

Japan

**Satoko Watanabe**

Japan

**Stephen Weeks**

Belgium

**Benedikt Wefer**

Germany

**Sven Wegerhoff**

Germany

**Roland Weis**

Austria

**Steve Whyard**

Canada

**Andrew Wilber**

USA

**David Wilson**

USA

**Somrutai Winichayakul**

New Zealand

**Gavin Wright**

UK

**Qiang Wu**

China

**Lian Xijun**

China

**Montarop Yamabhai**

Thailand

**Kenji Yamamoto**

Japan

**Boxu Yan**

USA

**Ming-Te Yang**

Taiwan

**Kechun Zhang**

USA

**Zhanying Zhang**

Australia

**Shoujing Zhao**

China

